# Reviewed article: Baggio AC, Mahana G, Oliveira C, Campos JC, Trevisol DJ, Schuelter-Trevisol F, Traebert E, Traebert J. Self-perception of depressive symptoms, self-reported medical diagnosis, and associated factors: findings from the Brazilian Longitudinal Study of Aging, 2019-2021. Epidemiol Serv Saude. 2026:35;e20250158

**DOI:** 10.1590/S2237-96222026v35e20250158.a

**Published:** 2026-01-16

**Authors:** Claudia de Souza Lopes

**Affiliations:** 1 Universidade do Estado do Rio de Janeiro, Rio de Janeiro, RJ, Brazil

## Peer review A

## Questionnaire

Does the manuscript contain new and significant information to justify publication? Yes

Does the Abstract (Summary) clearly and accurately describe the content of the article? No

Is the problem significant and concisely stated? No

Are the methods described comprehensively? No

Are the interpretations and conclusions justified by the results? No

Is adequate reference made to other work in the field? No

## Manuscript Structure

Length of article is: Adequate

Number of tables is: Adequate

Number of figures is: Adequate

Please state any conflict(s) of interest that you have in relation to the review of this paper. None

Interest: Good

Quality: Below Average

Originality: Average

Overall: Below Average

Recommendation: Major Revision

## Comments

O manuscrito aborda um tema relevante para a área da saúde coletiva e dos cuidados em saúde: a elevada prevalência de sintomas depressivos em idosos. O estudo utiliza dados transversais de um inquérito de base populacional de grande porte (ELSI - Brasil). No entanto, o manuscrito necessita de ampla revisão, pois apresenta trechos pouco claros, além de erros de conteúdo e forma. A seguir, detalho os principais pontos que devem ser revistos:

Referências:

O formato e a ordem das referências apresentam diversos erros. Por exemplo, a frase “Nesse contexto, a depressão é um agravo frequente em idosos, pois com o aumento da idade mais sintomas depressivos são apresentados, principalmente por meio de queixas relacionadas a diferentes comorbidades¹” apresenta como referência um artigo diferente daquele listado como (1) no início do texto. A revisão completa das referências é necessária.

Resumo:

1. Os objetivos estão mal formulados e devem ser apresentados no plural, pois envolvem mais de uma questão de pesquisa. Recomenda-se explicitar os fatores associados avaliados. Se a variável dependente é o autorrelato de diagnóstico médico de depressão, a estimativa de sua prevalência deve ser o ponto de partida. Sugestão:

Objetivos: Estimar a prevalência de autorrelato de diagnóstico médico de depressão e avaliar sua associação com autopercepção de sintomas depressivos (sugiro que isso seja feito), variáveis sociodemográficas, atividade física, consumo de bebida alcoólica e tabagismo.

2. A frase “Verificou-se associação de autorrelato de sintomas depressivos...” está incompleta - deve-se especificar com quais variáveis houve associação.

3. Reescrever conforme a sugestão:

Entre as mulheres, o autorrelato de diagnóstico médico de depressão foi cerca de duas vezes maior do que entre os homens, enquanto pessoas com escolaridade inferior a oito anos e que não realizavam atividade física apresentaram uma prevalência cerca de 30% maior do que aquelas com maior escolaridade e que praticavam atividade física.

4. Substituir o termo “testado” por “avaliado”.

5. A conclusão deve ir além da repetição dos resultados. Deve enfatizar os principais achados e, sobretudo, sua relevância para a saúde coletiva e os cuidados com a população idosa.

Introdução:

• A referência 1 (Maier et al., 2021) não sustenta adequadamente a afirmativa feita. Seria mais apropriado utilizar estudos populacionais que comparem a prevalência de depressão entre idosos e adultos.

• A frase “Por exemplo, alguns despertam sentimentos de preocupação excessiva, sensação de ninho vazio, alterações hormonais como menopausa nas mulheres ou fatores externos como aposentadoria podem desencadear uma maior complexidade dos casos (7)” deve ser reescrita para maior clareza e correção gramatical.

• O trecho que descreve critérios diagnósticos do TDM segundo o DSM-5 deve ser excluído, pois foge ao escopo do estudo, que trabalha com autorrelato de diagnóstico médico e autopercepção de sintomas, e não com diagnóstico clínico.

• Em seu lugar, é fundamental destacar a importância do uso do autorrelato como proxy em inquéritos epidemiológicos, incluindo suas limitações - como o viés relacionado ao acesso aos serviços de saúde.

• O trecho sobre fatores associados à resposta ao tratamento também deve ser retirado, por não fazer parte dos objetivos do estudo.

• A justificativa do estudo deve ser reformulada. É necessário apontar lacunas na literatura e como este estudo contribui para preenchê-las.

• Não há menção à literatura nacional que utilizou dados da PNS 2013 e 2019, que também empregou o autorrelato de diagnóstico e o PHQ-9 - essas referências devem ser incluídas.

• Os objetivos devem ser reescritos conforme sugerido no resumo.

Métodos:

1. O termo “epidemiológico” pode ser retirado, por ser redundante.

2. A dicotomização de variáveis de estilo de vida empobrece a análise. Se a base de dados permitir, sugere-se utilizar categorias mais detalhadas que reflitam melhor a heterogeneidade desses comportamentos.

3. Não está claro se foi avaliada a associação entre autopercepção de sintomas depressivos e o autorrelato de diagnóstico médico de depressão - isso deve ser esclarecido.

4. Sugiro incluir a associação da autopercepção de sintomas depressivos e autorrelato de diagnóstico médico de depressão.

Resultados:

1. Afirmar que os participantes não praticam atividades “ainda que leves” não é adequado, já que essa classificação não foi utilizada no estudo.

2. Tabela 1:

o Título sugerido: Prevalência de depressão segundo autorrelato de diagnóstico médico e sua associação com variáveis sociodemográficas, comportamentais e clínicas.

o Incluir o total de participantes (N).

o Remover a linha de total - os percentuais devem ser apresentados por categoria.

3. O mesmo vale para a Tabela 2.

4. Tabela 3: Especificar no rodapé quais variáveis foram incluídas no modelo ajustado. O texto menciona critério de p<0,20, mas há variáveis incluídas (como tabagismo) com p>0,5 - isso precisa ser justificado ou revisto.

Discussão:

1. Iniciar com um parágrafo que sintetize os principais achados do estudo.

2. Evitar afirmações como “abordagem pouco comum”, pois o uso de autorrelato é amplamente empregado em estudos epidemiológicos.

3. A discussão deve contextualizar os achados com base em estudos semelhantes, destacando convergências, divergências e possíveis explicações.

4. Trechos que justificam o uso do autorrelato devem ser deslocados para a introdução, caso mantidos.

Revisar integralmente manuscrito à luz das sugestões acima.

